# The impact of health literacy, patient-centered communication and shared decision-making on patients’ satisfaction with care received in German primary care practices

**DOI:** 10.1186/s12913-016-1693-y

**Published:** 2016-08-30

**Authors:** Sibel Vildan Altin, Stephanie Stock

**Affiliations:** Institute for Health Economics and Clinical Epidemiology, University Hospital of Cologne, Gleuelerstr. 176-178, 50935 Cologne, Germany

**Keywords:** Health literacy, Patient-centered communication, Shared decision-making, Patient satisfaction, Primary care, Patient-reported outcomes, Health literate health care organization

## Abstract

**Background:**

Findings on the association between health literacy skills and patient-reported outcomes such as satisfaction with health care delivery are scarce. We explored the extent to which subjective health literacy skills and the perception of the application of patient-centered communication and shared decision-making are associated with patient’s satisfaction with care received by their general practitioner (GP).

**Methods:**

A nationwide cross sectional survey was administered in a random sample of 1125 German adults. A binary logistic regression model controlling for demographics and health status was used to examine the independent contributions of predictor variables (i.e. subjective health literacy, shared decision-making, patient-centered communication) on satisfaction with care received by the GP.

**Results:**

Respondents with sufficient health literacy skills were 2.06 times as likely (95 % [CI]: 1.002–4.264) and those who were involved in shared decision-making by their GP were 4.02 times as likely (95 % [CI]: 1.849–8.744) to be satisfied with care received by their GP. Respondents who experienced that their GP explained things in an easy to understand way (OR: 4.44; 95 % [CI]: 1.817–10.869), knew important things about their medical history (OR: 3.46; 95 % [CI]: 1.502–7.994) and spent enough time with them, also reported to be more satisfied (OR: 3.12; 95 % [CI]: 1.410–6.905).

**Conclusion:**

German adults having sufficient subjective health literacy skills and experiencing a more patient-centered relationship with their GP are more likely to be satisfied with care. These findings are important for health care organizations aiming to respond to health literacy needs of patients.

**Electronic supplementary material:**

The online version of this article (doi:10.1186/s12913-016-1693-y) contains supplementary material, which is available to authorized users.

## Background

The provision of health care in a way that patients are empowered to understand and act upon crucial health information when making healthcare decisions is gaining in importance. This is due to the fact that various studies demonstrate a lack of health literacy skills in large parts of populations in Europe and the US contributing to undesirable outcomes such as a limited adherence to medication-regimes, insufficient self-management skills and more frequent hospitalizations and emergency care utilization [1, 2]. In this regard, evidence from Germany suggests that more than half of the population is affected by limited health literacy skills [1, 3].

To respond to the burden of limited health literacy, the Institute of Medicine (IOM) recently proposed to transform healthcare organizations to health literacy responsive ones by redesigning their structures and processes to support low literate patients to navigate, understand, and use information and services to take care of their health [4, 5]. According to the IOM report on the attributes of health literate healthcare organizations (HLHO), this transformation can be accomplished by encouraging health care organizations to implement elements of patient-centered care. In their concept, patient centered care is defined as “care that is respectful of and responsive to individual patient preferences, needs, and values” and that ensures “that patient values guide all clinical decisions” [6]. Therefore, the implementation of such care needs health care organizations and its staff to move back from disease oriented care and engage in a close patient-physician partnership trying to produce the best outcome possible for the patient [7]. This focus on the patient’s preferences and values also involves the consideration of patient’s resources and competencies to take part in their own health care, making it necessary to also focus on the health literacy responsiveness of health care organizations. In this regard, the implementation of a more patient-centered and health literacy responsive care takes many efforts on the side of health care organizations [8, 9]. One very meaningful measure is to enhance patient-centered communication by training health care professionals to use plain language, confirm understanding when communicating with the patient and secure language assistance for patients with foreign languages [8, 10, 11]. A further significant step is to enable shared decision-making, which is denoted as a process in which clinicians and patients share the best available evidence when making a health care decision. [12–14]. These elements can subsequently contribute positively to the health literacy responsiveness of a health care organization [15]. Investigating the evidence on health care experiences of low health literate populations substantiates the growing need to initiate the change process in health care organizations. In this regard, previous studies yield the finding that clinicians view low health literate populations as one of the most challenging patient groups to communicate effectively with and to involve in shared decision-making [16]. Concurrently there is evidence that enhancing patient-provider communication and shared decision-making results in greater patient satisfaction, adherence to treatment plans, and improved health outcomes [17]. Studies investigating the interrelations between patient-centered communication, shared decision-making and health literacy demonstrate that low health literate patients are less likely to experience participatory decision making in the clinical encounter [18] and more likely to perceive interpersonal communication with health care professionals as less patient-centered [19, 20]. Studies examining the impact of both, perceived shared decision-making and patient-centered communication on patient-reported health care experiences among low literate populations are scarce [21]. However, there is a growing need to better understand the effects of personal care experiences (e.g. patient centered care) and patient specific factors (e.g. socioeconomic status, health beliefs, health literacy) on patient reported outcomes on the quality of health care delivery (e.g. satisfaction with care). Better insights in these interrelations would allow the development of measures to initiate organizational change towards a more patient centered and health literacy friendly environment. The current lack of evidence on the effects of limited health literacy and health care experiences on outcomes such as perceived health care quality is likely to contribute to the slow diffusion of the concept of health literate health care organizations in health policy agendas and health care organizations [22]. However, organizational change is particularly relevant for primary care which is regarded as a meaningful setting to diminish the literacy related inequalities in health care [23, 24]. Nevertheless, insights into the importance of limited health literacy for patient satisfaction in primary care are scarce and not consistent, so far [25]. In order to close the lack in knowledge, we performed a nationwide representative survey among the German adult population and explored if subjective health literacy skills and the perception of the application of patient-centered communication and shared-decision making in primary care is associated with patients satisfaction with the care received by their personal general practitioner (GP).

We hypothesize that care experiences in shared decision-making and patient-centered care as well as the subjective health literacy level will be independently associated with patient’s satisfaction with care delivery.

## Methods

### Study design and participants

This study is a cross sectional survey involving computer-assisted telephone interviews with a nationally representative random sample of adults aged 18 and older living in Germany. Data was derived from the 2013 Commonwealth Fund International Health Policy Survey. The survey was carried out by the social science company Social Science Research Solutions (SSRS) and the BQS Institute for Quality and Safety. The sample was contacted from February to May 2013 by random-digit dialing of both landlines and mobile phones throughout Germany. Up to eight calls were made to establish contact. Interviewers ascertained whether there were residents in the household within the age range and, if there were multiple, they selected one for the interview using the nearest birthday method. The margin sample error was approximately plus or minus 3 % for the sample. The response rate was 11.0 %, defined as completed interviews (*N* = 1125) out of the overall sample members that could be contacted (*N* = 10.300). Results were weighted to be nationally representative using data on age, sex, region, and education. Differential nonresponse was also addressed through weighting to provide nationally representative findings. Full details of the methodology used for the 2013 Commonwealth Fund International Health Policy Survey have been published previously [26]. This cross sectional survey has been performed in accordance with the Declaration of Helsinki. Due to the fact that the survey was non-medical ethical approval from the Regional Ethical Review Board was not required [27]. Participation in the survey was voluntary and verbal informed consent was obtained from every participant before the questionnaire was answered. Confidentiality was maintained by data coding to eliminate the identification of data with personal information. This study follows the STROBE guideline for reporting of observational studies. An additional file shows the completed STROBE checklist [see Additional file 1].

### Study variables

#### Demographics

Patient demographic information included age, gender, educational attainment (low, middle, high educated), migrant status and insurance type (public/private). Educational attainment was categorized according to the International Standard Classification of Education (ISCED) organizing educational attainment in three levels (low, middle and high education) [28].

#### Health status

Self-rated health status was assessed using one item asking “In general, how would you describe your own health?”. The item is answered on a five point Likert scale ranging from “poor” to “excellent”. In addition, a more objective indicator was added asking if someone was diagnosed with a chronic condition such as diabetes, coronary artery disease, hypertension, asthma, or a depression. For the analysis self-rated health status was analyzed as a binary outcome (1 = fair/poor; 0 = excellent, very good, good).

#### Health literacy

Health literacy was measured using a one-item screener retrieved from the Brief Health Literacy Screen (BHLS), a verbally administered self-report measure of subjective functional health literacy. The screener item was developed by Chew and colleagues and has been validated against widely used measures of health literacy [29, 30] across a variety of settings [31–33]. The respondents were asked: “How often do you have problems learning about your medical conditions because of difficulty understanding written information?”. The item is answered on a five point Likert scale ranging from “always” to “never”. Following prior studies, we coded respondents who reported to have “rarely/never” problems learning about their medical condition as having “no problem” and respondents who reported to have “always/often” problems as having a “problem”. “Don’t know” responses were considered as missing.

### Experiences with healthcare delivery in the general practice setting

#### Patient-centered communication

To assess the perceived patient-centeredness of communication in the general practice from the patient perspective, we used the three best performing items from the health care provider communication subscale of the validated Consumer Assessment of Healthcare Provider and Systems (CAHPS) clinician and group survey [34–36]. The 6 item subscale assesses the health literacy related aspects of interpersonal communication and navigation assistance from the patient perspective especially emphasizing the actual application of health literate communication strategies through healthcare professionals. The respondents were asked: “How often does your general practitioner or medical staff you see: (1) …explain things in a way that is easy to understand? (2) …know important information about your medical history? (3) …spend enough time with you?”. Each of the CAHPS items was answered on a four point Likert scale ranging from “always” to “never”. The items were analyzed as a binary outcome (1 = never/rarely; 0 = always/often).

#### Shared decision-making

Perceived shared decision-making was assessed by using an item developed by the Commonwealth Fund and used in the longitudinal International Health Policy Survey of Commonwealth Fund since 2011 [37]. The respondent was asked: How often does your general practitioner or medical staff you see involve you as much as you want to be in decisions about your treatment or care?”. The item was answered on a four point Likert scale ranging from “always” to “never”. The item was analyzed as a binary outcome (1 = never/rarely; 0 = always/often).

#### Satisfaction with the care received in the general practice

Perceived satisfaction with the care received by the general practitioner was measured using the item „How do you rate the overall medical care received in the last 12 months by your general practitioner?”. The item was developed by the Commonwealth Fund and used in the longitudinal International Health Policy Survey of Commonwealth Fund since 2010 [38]. Response was assessed on a four point Likert scale ranging from “1 = poor” to “4 = very good”. The item was analyzed as a binary outcome (1 = very good, good; 0 = fair, poor). Since nearly all respondents have a personal GP they consult on a regular basis, the measurement approach for the outcome variable, asking to think of care received by the personal GP in the last 12 months is appropriate.

### Statistical analysis

Demographic data was analyzed using means, frequencies, and cross tabulations to calculate descriptive statistics. Associations between the outcome (perceived satisfaction with care received by the general practitioner) and predictor variables (i.e. patient-centered communication, shared decision-making, health literacy) as well as between the predictor variables were examined by conducting bivariate analysis using chi-squared tests for independence. The main study hypothesis was examined by applying binary logistic regression analyses. The dichotomized item assessing the satisfaction with the care received by the general practitioner served as the dependent variable. The model was controlled for age, gender, educational attainment, migration status and self-rated health. Missing values for a variable were not included in analysis using that variable. Data was analyzed with SPSS version 21. Statistical significance was assessed as *p* < 0.05.

## Results

### Participant characteristics

Characteristics of our survey sample are described in Table 1. Survey participants were in average 52.4 years old, 60 % were female and 43.6 % had a high school education or less. Health status variables indicated that about 76.0 % of the sample had a good to very good health whereas 31.8 % was affected by two or more chronic conditions with hypertension and coronary artery disease being the most prevalent. Almost all respondents, (94.8 %) did have access to a general practice they consulted on a regular basis.

**Table 1:** Demographic characteristics

Variable	Number	Percent
Participants	1125	
Age		
Mean ± SD	52.43 ± 17.73	
Range	18 – 96	
18–34 years	227	20.2
35–49 years	262	23.3
50–64 years	330	29.3
≥ 65	306	27.2
Gender		
Female	680	60.4
Male	445	39.6
Migration status		
Non-migrant	921	82.4
Migrant	197	17.6
Education degree		
Middle school degree	192	17.1
Intermediate high school	298	26.5
degree	254	22.6
University entrance qualification	195	17.3
Insurance status		
Statutory health insurance	963	85.6
Private insurance	151	13.4
*Primary care access status*		
Personal general practitioner	1066	94.8
Overall health status		
Very good	408	36.3
Good	444	39.5
Fair	202	18.0
Poor	61	5.4
Chronic conditions		
< 2 chronic conditions	715	68.2
≥ 2 chronic conditions	334	31.8
Diabetes	99	8.8
Coronary artery disease	143	12.7
Hypertension	349	31.0
Asthma	114	10.1
Depression	134	11.9

### Predictors of satisfaction with care received by the general practitioner

As presented in Table 2, in bivariate analysis, both the perceived application of patient-centered communication as well the use of shared decision-making in consultations with the GP were positively associated with satisfaction with care received by the GP in the last 12 months (*p* < 0.001). Sufficient health literacy skills were also positively related to reporting satisfaction with the care received by the GP (*p* < 0.05). There was no statistically significant association between patient-centered communication, shared decision-making and health literacy.

**Table 2:** Bivariate associations between patients health literacy skills, communication and shared decision-making experiences in primary care and patients satisfaction with their general practitioner (*N* = 1038)

Variable	Satisfaction with the care received by the general practitioner in the last 12 months (very good, good)		
	*N*	(%)	*p*-value^*^
Patient-centered communication			
How often does your general practitioner or medical staff you see …			
Explain things in a way that is easy to understand			<0.001
always/often	910	94.3	
never/rarely/sometimes	36	54.5	
Know important information about your medical history			<0.001
always/often	865	93.7	
never/rarely/sometimes	56	69.1	
Spend enough time with you			<0.001
always/often	851	94.2	
never/rarely/sometimes	92	74.8	
Shared decision-making			
Involve you as much as you want to be in decisions about your treatment or care			<0.001
always/often	837	94.3	
never/rarely/sometimes	76	71.0	
Health Literacy			
Problems when learning about medical conditions			<0.05
no problem (rarely/never)	589	93.5	
problem (always/often)	160	88.4	

^*^ The *x* ^*2*^ test for independence was used for categorical variables

In the binary logistic regression model, experiencing a rather patient-centered communication in the general practice and being involved in shared decision-making with the GP as well as having less problems understanding written information when learning about own medical conditions were significant independent predictors of being satisfied with the care received in the general practice (Table 3). Respondents who experienced that their GP explained things in an easy to understand way were 4.44 times as likely (95 % CI [1.817 – 10.869] *p* < 0.001) to report to be satisfied with the care they received in the general practice they consulted on a regular basis in the last 12 months. Respondents who reported that their GP knew important things about their medical history were 3.46 times as likely (95 % CI [1.502 – 7.994] *p* < 0.01) to be satisfied with care. Respondents who experienced that their GP often or always spend enough time with them also reported to be satisfied with their GP (OR: 3.12; 95 % CI [1.410 – 6.905] *p* < 0.01). Respondents who experienced that their GP often or always involved them in decisions about their treatment or care as much as they desired were 4.02 times as likely (95 % CI [1.849 – 8.744] *p* < 0.001) to report a high satisfaction with the care received. Respondents with adequate health literacy skills in terms of having less problems understanding written information when learning about their own medical conditions were 2.06 times as likely (95 % CI [1.002 – 4.264] *p* < 0.05) to report to be highly satisfied with the care received in the general practice of their personal GP in the last 12 months.

**Table 3:** Predictors of satisfaction with the care received by the general practitioner in the last 12 months in a binary logistic regression model (*N* = 703)

Variable	Satisfaction with the care received by the general practitioner in the last 12 months (very good, good)		
		95 %-CI for EXP (B)	
	Odds ratio	Lower	Upper
Predictor variables			
Patient-centered communication			
How often does your general practitioner or medical staff you see …			
Explain things in a way that is easy to understand? ^a^			
always/often	4.444***	1.817	10.869
Knows important information about your medical history? ^a^			
always/often	3.466**	1.502	7.994
Spend enough time with you? ^a^			
always/often	3.120**	1.410	6.905
Shared-decision making			
Involve you as much as you want to be in decisions about your treatment or care? ^a^			
always/often	4.021***	1.849	8.744
Health Literacy			
How often do you have problems learning about your medical conditions because of difficulty understanding written information? ^b^			
no problem (rarely/never)	2.067*	1.002	4.264
Sociodemographic covariates			
Age ^c^			
35–49 years	1.575	0.626	3.996
50–64 years	1.938	0.743	5.056
≥ 65 years	2.217	0.827	5.941
Education status ^d^			
Middle educational status	1.044	0.485	2.247
High educational status	0.937	0.373	2.353
Gender			
Male	1.428	0.678	3.007
Migration status			
Migrant	0.683	0.316	1.478
Health related covariates			
Health status ^e^			
Excellent/good	3.691***	1.759	7.745

*p < 0.05. **p < 0.01. ***p < 0.001

^a^ Referent category is never/rarely/sometimes

^b^ Referent category is problematic (always/often)

^c^ Referent category is 18–34 years

^d^ Referent category is low educational status

^e^ Referent category is fair/poor

In our model, self-reported health was a significant covariate, thus respondents with excellent or good health were more likely to report to be highly satisfied with the care received by the GP compared to respondents with rather fair or poor health. Socio-demographic variables were not significantly associated with the outcome. To support the reliability of our model to detect the independent effect of the predictor variables, we performed a post hoc power analysis. For our dependent variable, given the sample size of *N* = 703 and setting a type 1 error (α) at 0.05 (two-sided), we had an 80 % power to detect an effect size of 0.15. Therefore our study was sufficiently powered.

## Discussion

Patient satisfaction with care is an important indicator of process of care and is a common marker for quality of care [39]. By now, insights in the interrelations of patient’s health literacy and satisfaction with primary care are scarce [25]. Given this, our results of a nationwide representative survey of German adults indicate that the perceived application of patient-centered communication and the involvement of the patient in shared decision-making are both independently associated with patient’s perceived satisfaction with care received in the primary care practice. Respondent’s ability to understand written information when learning about the own medical condition is also significantly associated with perceived satisfaction with care received in primary care. Further, there is no significant association between patient-centered communication, shared decision-making and health literacy. The overall results indicate that apart from a satisfactory interpersonal patient-physician relationship, the aspect of sufficient health literacy has also a relevant impact on the perception of quality of care in terms of satisfaction with health care delivery in the primary care setting.

Our findings are novel since insights into the association between health literacy, perceived quality of interpersonal communication and patient reported outcomes are limited and previous findings are rather inconclusive [19, 21, 40]. In contrast to the somewhat more explicit links between patient-centered communication as well as shared decision-making and satisfaction with care indicating a positive association [17, 41, 42], the few studies examining the association between health literacy and satisfaction with primary care yield a rather weak association [25]. A closer look on the association between limited health literacy and the perceived application of shared decision-making and patient-centered communication also reveals a positive association indicating that patients with self-reported health literacy challenges experience less participatory decision-making and perceive interpersonal communication with health care professionals as less patient-centered [18–20]. Our findings in the primary care setting do not support prior findings on interrelations between health literacy, patient centered-communication and shared decision-making. But they do verify prior findings on the interrelations between shared-decision making, patient-centered communication and satisfaction with care and expand current knowledge regarding the additional association between health literacy and perceived quality of care in the general practice.

Our findings illustrate significant implications for the future development of health literate health care organizations, since our results demonstrate that health literacy level is associated with patient reported outcomes such as patient satisfaction with care. Therefore, organizations intending to implement structures and processes that empower patients to take care of their health should be encouraged to support patients to navigate, understand, and use health information and services. To support patients with limited health literacy, health care organizations can engage in the implementation of specific interventions that target the improvement of patient’s health literacy skills or help to respond to the health literacy needs of patients. Several scholars and organizations such as the WHO and IOM already propose organizational techniques deemed helpful to identify and support patients with particular health literacy needs [5, 43, 44]. Further, a recent review on purposeful interventions to address the issue of limited patient health literacy conducted by the Commonwealth Fund, also recommends specific measures for primary care [45]. The existing action plans and manuals for health care organizations encompass recommendations on oral and written communication as well as supportive actions to help patients organize their care [44]. Effective interventions on patient-physician communication involve the implementation of trainings for health care staff equipping them with competencies to use standardized communication tools, such as the teach back (checking patient understanding by letting the patient explain medical issues in his own words) and chunk and choke method (breacking down information into manageable sections and checking patient understanding) and the use of plain language without medical jargon [46, 47]. Interventions involving written information encompass the use of audio/visual health information and pictorials as well as educational materials adjusted to the reading level of low literate patients (e.g. fact boxes, decision aids) [44, 48, 49]. Supportive measures to help patients organize their care and navigate through the health care system involve the coordination of medical appointments by health care staff, application of follow up services to check the therapy adherence of patients, use of medication reviews to check medication adherence, application of therapy plans with easy understandable written information for patients [46].

In addition, there are also national actions plans and policy roadmaps in the US, Australia and European Union available that inform policy and decision makers on suitable national strategies to improve the population health literacy [43, 50, 51]. These action plans include measures for the educational, health care and workplace setting.

Therefore, our results highlight the importance of the implementation of interventions to initiate the change process towards a health literate health care organization, going beyond the perceived application of patient-centred communication and participative health decision-making. Within this change process, aspects of organizational and behavioural change need to be considered when choosing and implementing interventions [5, 8]. In this regard scholars highlight that it is from major importance to not just implement certain interventions but also to take into account measures to change the organizational culture and aims towards a more patient-centered understanding of care [52, 53]. Therefore aspects of professional roles of health care practitioners as well as leadership structures need to be considered to make changes sustainable [54]. Such aspects could be addressed by using change agents who supervise staff in the application of interventions to address limited heath literacy or by implementing standard operating procedures for health literacy interventions that are combined with financial incentives [52, 53]. A comparable and recent organizational change process that could serve as a role model here is the patient safety and quality assurance agenda setting the stage in developed countries [55].

Our study has certain strengths that need to be noted. The data used is nationally representative for the German adult population. Moreover, the development of the survey items followed a scientifically rigorous process including cognitive tests of the outcome item and the use of validated instruments to measure health literacy and patient-centered communication. In regard to the limitations of our study, there are also specific aspects that need to be mentioned. Although patient self-report of perceived health care quality is a valuable way to assess the quality of care, objective measures are preferable [56]. From a methodological perspective, it should be mentioned that the low response rate of 11.0 % suggests a potential nonresponse-bias, although the direction of that bias is unknown. In the case of our study, it might be that elderly with limited health literacy skills may try to hide their difficulties [57] and be less likely to take part in surveys [58]. In this regard, there is evidence that people with poor literacy and a low socio-economic status as well as members of minority ethnic groups and the elderly are groups which are hard to reach in social science research [59, 60]. Therefore, there is potential that our study overlooked especially these groups. One additional reason for the high non-response rate might be the rapid response design of the survey with a field time of eight weeks. However, it needs to be pointed out, that interviewers called potential survey participants at least eight times if they did not receive a response.

Future research should deepen the insights into the interrelations between health literacy, patient-centered care, shared decision-making and health care quality investigating the causal pathways and underlying factors affecting the associations.

## Conclusion

German adults having sufficient health literacy skills and experiencing a more patient-centered communication and involvement in shared decision-making by their GP are more likely to be satisfied with the care received in the general practice compared to Germans with limited health literacy skills and poor experiences in interpersonal communication and shared decision-making. This result is from major importance for health care organizations aiming to implement processes and structures to become more responsive to the health literacy needs of their patients, emphasizing that a successful implementation of a HLHO requires the application of a comprehensive strategy going beyond the application of patient-centred communication and participative health decision-making.

## Additional file

Additional file 1: STROBE Statement—Checklist of items that should be included in reports of *cross-sectional studies.* (DOCX 40 kb)

## Abbreviations

BHLS: Brief health literacy screen
BQS: Institute for quality and safety
CAHPS: Consumer assessment of healthcare provider and systems
GP: General practitioner
HLHO: Health literate health care organization
IOM: Institute of medicine
ISCED: International standard classification of education
SSRS: Social science research solutions
